# Detection of *Burkholderia pseudomallei* toxin-mediated inhibition of protein synthesis using a *Caenorhabditis elegans ugt–29* biosensor

**DOI:** 10.1038/srep27475

**Published:** 2016-06-07

**Authors:** Rui-Rui Wong, Cin Kong, Song-Hua Lee, Sheila Nathan

**Affiliations:** 1 School of Biosciences and Biotechnology, Faculty of Science and Technology, Universiti Kebangsaan Malaysia, 43600 UKM Bangi Selangor, Malaysia; 2 Malaysia Genome Institute, Jalan Bangi, 43000 Kajang, Selangor, Malaysia

## Abstract

Toxins are believed to play a crucial role in *Burkholderia pseudomallei* pathogenicity, however to date, only a few have been identified. The discovery of additional toxic molecules is limited by the lack of a sensitive indicator of *B. pseudomallei* toxicity. Previously, from a whole genome transcriptome analysis of *B. pseudomallei*-infected *Caenorhabditis elegans*, we noted significant overexpression of a number of worm genes encoding detoxification enzymes, indicating the host’s attempt to clear bacterial toxic molecules. One of these genes, *ugt–29*, a family member of UDP-glucuronosyltransferases, was the most robustly induced phase II detoxification gene. In this study, we show that strong induction of *ugt–29* is restricted to infections by the most virulent species among the pathogens tested. We also noted that *ugt–29* is activated upon disruption of host protein synthesis. Hence, we propose that UGT–29 could be a promising biosensor to detect *B. pseudomallei* toxins that compromise host protein synthesis. The identification of bactobolin, a polyketide-peptide hybrid molecule, as a toxic molecule of *B. pseudomallei* further verifies the utilization of this surveillance system to search for bacterial toxins. Hence, a *ugt–29* based reporter should be useful in screening for other molecules that inhibit host protein synthesis.

*Burkholderia pseudomallei,* a Gram-negative soil bacterium, is the causative agent of melioidosis, an endemic disease which results in high mortality of infected humans^1^,^2^. In Northeast Thailand, the number of deaths from melioidosis is the third highest after HIV-acquired immunodeficiency syndrome and tuberculosis^3^. Despite extensive studies undertaken to unravel the pathogenesis during the establishment of infection, many of the *B. pseudomallei* virulence factors still remain uncharacterized.

In an earlier study, we had analyzed the genome-wide transcriptome profile of *B. pseudomallei* infected *Caenorhabditis elegans* to elucidate host responses to the infection within the context of a whole animal^4^. Approximately 6% of the worm genome was modulated during the infection and amongst the genes that were robustly induced were members of a detoxification enzyme family, proposing the importance of bacterial toxins in the pathogenesis of *B. pseudomallei*. Previously, O’ Quinn *et al*.^5^ suggested that *B. pseudomallei* produces a paralytic endotoxin which leads to the perturbation of Ca^2+^ homeostasis and neuromuscular intoxication in worms. This corresponds to the disease manifestation in higher order animals where paraparesis is reported as a prominent neurological presentation of melioidosis in mice, goats and sheep^6^,^7^. In addition, Ooi *et al*.^8^ systematically demonstrated that unlike other well-studied pathogens, *B. pseudomallei* does not colonize the worm intestinal lumen during infection. In light of the rapid death of *B. pseudomallei* infected worms, it is very likely that *B. pseudomallei* adopts toxin-mediated killing as the major virulence mechanism in maintaining an active infection and contributing to the death of the infected nematode. However, to date, only limited *B. pseudomallei* toxic molecules have been identified, for example, *Burkholderia* Lethal Factor 1 (BLF1), a major toxin secreted by this bacterium^9^.

The difficulty in identifying the toxin(s) is in part due to the lack of a sensitive indicator to screen for *B. pseudomallei* toxicity. In toxicology studies, transgenic *C. elegans* have been widely utilized as nematode biosensors of xenobiotic chemicals from food and the environment^10^,^11^,^12^,^13^,^14^. Hence, we propose that utilizing transgenic worms as a biological sensor is useful to detect and predict additional toxic substances of *B. pseudomallei*. From the study on *C. elegans* response to a *B. pseudomallei* infection, the *ugt–29* gene was the most robustly up-regulated phase II detoxification enzyme-encoding gene where an induction of 77–fold was noted in *B. pseudomallei-*infected worms at 12 hours post infection^4^. As the resulting glycosylated derivatives of bacterial toxins are known to be less toxic, UDP-Glucuronosyl Transferase (UGT) is thought to play an active role in neutralizing bacterial toxins^15^.

In this study, we constructed a *C. elegans ugt–29* Green Fluorescence Protein (*ugt–29::GFP*) reporter and demonstrated that strong *ugt–29* expression is specific to worm infections by *B. pseudomallei* and the highly virulent *B. cepacia*. We show that the induction of *ugt–29* by *B. pseudomallei* requires activation of the cellular damage-response bZIP transcription factor, ZIP–2, that provides resistance to killing by *B. pseudomallei.* Based on these findings, we propose that host cellular impairment is an important consequence of a *B. pseudomallei* infection. RNAi-induced knockdown of genes involved in protein translation triggered the expression of *ugt–29*, suggesting that *zip-2*/*ugt–29* is surveillance-activated. Together, these findings imply that the expression of *ugt–29* is an adaptive transcriptional response toward a cellular defect caused by *B. pseudomallei* virulence factors. By using the *zip−2/ugt*−*29* surveillance system, we identified bactobolin as a toxic effector molecule that triggers a defect in host protein translation.

## Results

### *ugt-29* is expressed ubiquitously upon *B. pseudomallei* infection

**Figure 1:**
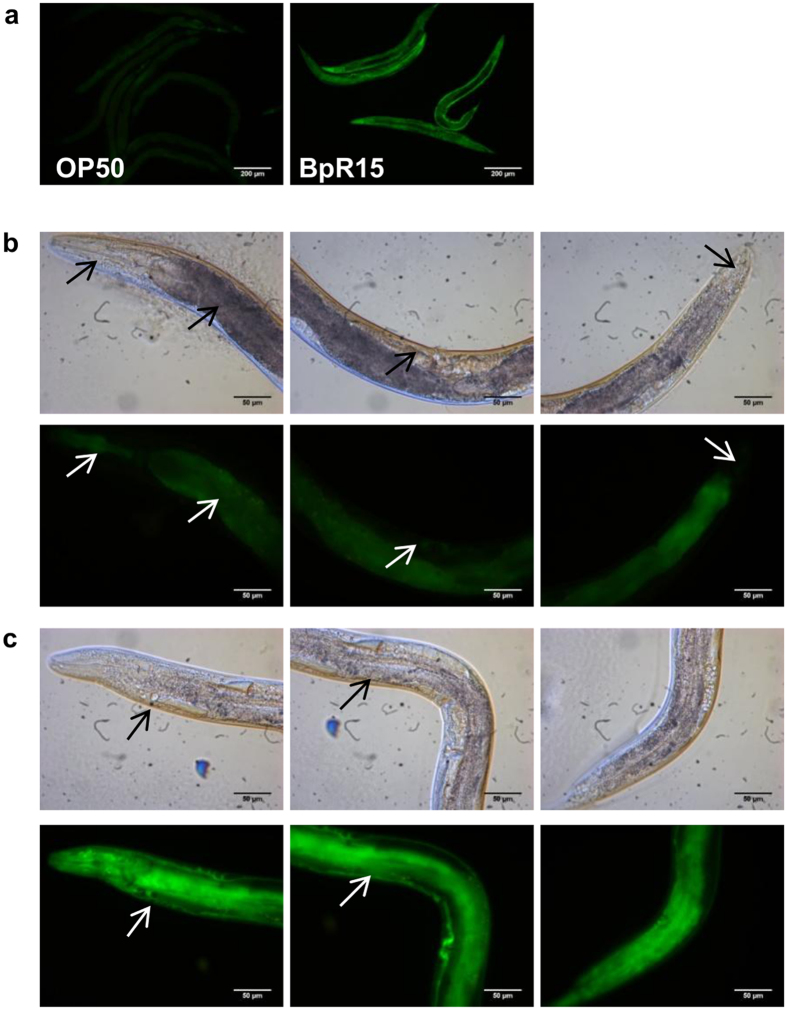
*ugt-29* is expressed ubiquitously in worms upon *B. pseudomallei* infection. (**a**) Representative fluorescence micrographs (100× magnification) of GFP expression indicating the endogenous transcriptional activity of *ugt–29* in worms fed with *E. coli* OP50 and BpR15 at 24 hours post infection. Representative micrographs (400× magnification) showing the basal expression of *ugt-29* in (**b**) *E. coli* OP50-fed worms and the elevated expression of GFP in (**c**) the worms infected by BpR15 at 8 hours post infection. For (**b**,**c**), micrographs in the upper panel are DIC images whilst the lower panel displays the fluorescence micrographs. Constitutive but dim expression of GFP was noted specifically at the pharynx, intestine, vulva muscle and tail (the body parts are denoted by arrows from left to right) of *E. coli* OP50-fed worms. Fluorescence micrographs of the same magnification were acquired at same exposure time and gain factor. Scale bars, 200 μm (100×) and 50 μm (400×).

To monitor *ugt–29* gene expression patterns in a whole animal, we constructed a transcriptional *ugt–29::GFP* reporter. The GFP reporter gene was fused to the *ugt–29* promoter, microinjected into a *pha–1(e2123)ts* mutant and maintained extrachromosomally. The pBX plasmid carrying a wild type copy of *pha-1* was co-injected to rescue the transgenic worms from embryonic lethality of the *pha–1* mutant at 25 °C^16^. Under 100 × magnification, *ugt–29::GFP* transgenic worms exhibited very dim GFP signals when fed on *Escherichia coli* strain OP50, the standard laboratory food for *C. elegans* (Fig. 1a). In contrast, the *ugt–29::GFP* transgene was robustly induced throughout the entire BpR15-infected worm over the period of infection (Supplementary Fig. S1). Next, the tissue distribution of *ugt–29* expression was observed using a higher magnification of 400×. As shown in Fig. 1b, constitutive but dim expression of GFP in the uninfected worms was evident specifically at the pharynx, intestine, vulva muscle and tail. In BpR15–infected worms, fluorescence was localized to the same tissues although at a much higher intensity. In addition, intense green fluorescence was also observed at the head and body wall muscle of BpR15-infected worms (Fig. 1c, lower panel).

### Robust *ugt-29* expression is specific to infections by virulent *Burkholderia* species and strains

**Figure 2:**
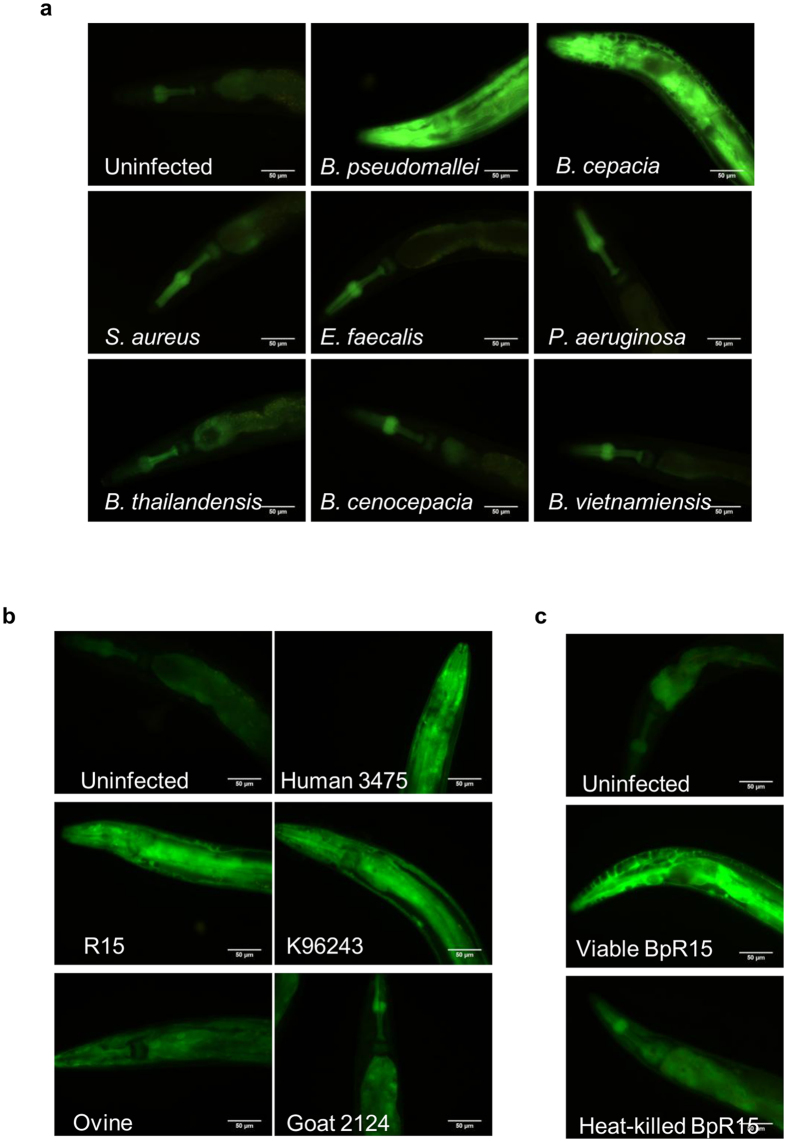
Strong ugt-29 expression is specific to infections by virulent *Burkholderia* species and strains. Representative fluorescence micrographs (400× magnification) of *ugt–29::GFP* reporter worms exposed to (**a**) various pathogens at 24 hours post infection, (**b**) individual *B. pseudomallei* isolates at 8 hours post infection and (**c**) heat-killed BpR15 relative to viable BpR15 at 24 hours post infection. All micrographs were acquired at same exposure time and gain factor. Scale bar, 50 μm.

To determine whether other bacterial pathogens induce *ugt–29*, we exposed the *ugt–29::GFP* transgenic worms to four other *Burkholderia* species (*B. thailandensis, B. cepacia, B. vietnamiensis* and *B. cenocepacia*) and three non-*Burkholderia* pathogens (*Pseudomonas aeruginosa, Staphylococcus aureus* and *Enterococcus faecalis*). Aside from *B. pseudomallei, B. cepacia* was the only pathogen able to induce robust *ugt–29::GFP* expression (Fig. 2a). Interestingly, data on the mean-time-to-death (TD_mean_) of nematodes infected by this cohort of bacterial pathogens demonstrated that *B. pseudomallei* and *B. cepacia* were significantly more virulent (*p* < 0.0001, log-rank test) towards the nematodes (Supplementary Fig. S2a). In worms fed with the other bacteria, delayed killing kinetics correlated with mild or negligible *ugt–29* expression as indicated by the similar GFP intensity between infected and uninfected worms. The fluorescence profile was consistent with the quantification of endogenous *ugt–29* expression by qRT-PCR (Supplementary Fig. S2b). Nevertheless, the observed *ugt–29* overexpression might represent changes due to the deadly consequences following infection and may be less apparent at this early time point in worm infections that present with a longer TD_mean_. Hence, in worms infected by *P. aeruginosa, S. aureus* and *E. faecalis*, we observed for fluorescence at a later stage of infection when ~50% of the worm population was killed. Again, we noted no significant increase in GFP intensity in infected worms at this later time point (Supplementary Fig. S3), suggesting that the induction of *ugt–29* is a specialized host response to specific infections rather than a generic response towards reduced nematode fitness following infection.

To further delineate the association between *ugt–29* expression and bacterial virulence, we challenged *ugt–29::GFP* transgenic worms with five different *B. pseudomallei* of varying virulence capacity. When compared with basal fluorescence in uninfected worms, infections with all five *B. pseudomallei* isolates triggered an observable increase in fluorescence expression, albeit the increase was relatively mild in worms infected by Ovine 3470 (TD_mean_ of 55.44 ± 2.163 hours) and Goat 2124 (TD_mean_ of 69.23 ± 1.097 hours) which were the two least virulent isolates tested (Fig. 2b, see also Supplementary Fig. S1c). Similarly, quantification of endogenous *ugt–29* expression by RT-PCR also reinforced the association between *ugt–29* overexpression and *B. pseudomallei* virulence (Supplementary Fig. S1d). Of note, the most virulent strain, Human 3475, triggered markedly enhanced transcription of *ugt–29* (134–fold) in worms following infection. In addition, unlike viable BpR15, heat-killed BpR15 which is not pathogenic to worms^17^ was not able to trigger robust *ugt–29* expression, again implying that induction of *ugt–29* is pathogenicity-related (Fig. 2c).

**Figure 3:**
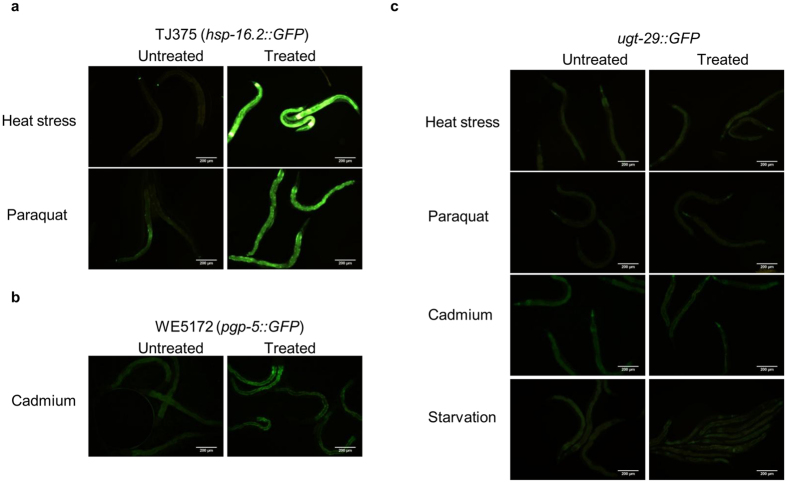
The expression of *ugt-29* was not visibly induced upon challenge by environmental stressors. GFP expression was observed in (**a**) TJ375 worms exposed to heat stress (37 °C for 1 hour) and 10 mM paraquat; (**b**) WE5172 worms exposed to 100 μM cadmium and (**c**) *ugt–29*::*GFP* worms exposed to heat stress, paraquat, cadmium and starvation. Paraquat-treated worms were examined at 6 hours post treatment whilst for the other three stress conditions, worms were assessed at 24 hours post treatment. The exposure time and gain factor are similar for all the micrographs taken. Scale bar, 200 μm.

Next, we asked if other abiotic stressors also induce similar robust expression of *ugt–29. ugt–29::GFP* worms were exposed to three different stressors (heat shock, paraquat and the heavy metal cadmium) and GFP fluorescence intensities were assessed relative to untreated worms. The transgenic worms carrying the *hsp–16.2::GFP* or *pgp–5::GFP* transgene were used as controls and exposed to heat shock and cadmium, respectively. As expected, both control worms exhibited an increase in GFP expression relative to untreated worms (Fig. 3a). In contrast, no observable increase in GFP expression was observed in *ugt–29::GFP* worms under all three stress conditions tested (Fig. 3b). In addition, we eliminated the possibility that the induction of *ugt–29* is a consequence of food limitation as a result of pathogen avoidance by *C. elegans* during a *B. pseudomallei* infection. GFP intensities were similar between starved and *E. coli* OP50-fed worms (Fig. 3b, lowest panel). Taken together, these observations suggest that *ugt–29* expression is likely a specialized response rather than a general stress response to *B. pseudomallei* infection.

### The conserved *zip–2* pathway is required for *ugt-29* induction upon *B. pseudomallei* infection

When confronted with infecting pathogens, *C. elegans* activates transcriptional responses that involve cooperative induction of innate immune effectors and detoxification genes to protect itself^4^,^18^,^19^. The distinct and robust expression of *ugt–29* in BpR15-infected worms suggests a prominent role of this gene in defense against *B. pseudomallei* infection. When we reexamined the cohort of *C. elegans* genes modulated during the *B. pseudomallei* infection, we noted that a number of the genes including *ugt–29* are known to be regulated by ZIP-2 in worms infected by *P. aeruginosa*^4^,^20^. In BpR15-infected worms, this cohort of genes was amongst those with the highest magnitude of induction^4^. As such, it is likely that ZIP-2 plays an important role in worm defense during a *B. pseudomallei* infection.

**Figure 4:**
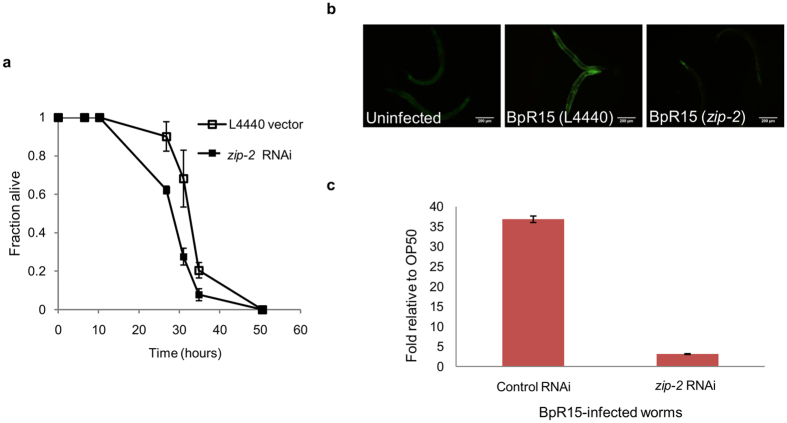
ZIP–2 is required to protect *C. elegans* from *B. pseudomallei* killing and to regulate the overexpression of *ugt-29* in *B. pseudomallei* infected worms. (**a**) Graph denotes the killing kinetics of worms infected by BpR15 upon *zip–2* RNAi knockdown. *zip–2* knockdown worms exhibited increased susceptibility to *B. pseudomallei* infection (*p* < 0.0001 log-rank test). Error bars represent mean value ± SD. Shown is the representative of two independent experiments (n = 120). (**b**) Representative fluorescence micrographs (100× magnification) of BpR15–infected *ugt–29::GFP* reporter worms upon *zip–2* RNAi knockdown at 6 hours post infection. The exposure time and gain factor are similar for all the micrographs taken. Scale bar, 200 μm. (**c**) qRT-PCR analysis of *ugt-29* in BpR15-infected worms upon *zip–2* RNAi knockdown at 6 hours post infection. Error bars represent mean values ± SD.

To confirm if ZIP–2 does indeed contribute to increased survival following a *B. pseudomallei* infection, we abolished the *zip–2* signaling pathway in *rrf–3(pk1426);glp–4(bn2)* double mutant worms and evaluated the *zip–2* RNAi worms susceptibility to *B. pseudomallei*. When the survival rate of the infected worms was compared to the control RNAi worms, we noted a significant reduction in *zip–2* RNAi worm survival (Fig. 4a). This verified that ZIP–2 is required for resistance against the killing effects of *B. pseudomallei*. Next, we asked if *ugt–29* is a downstream target of ZIP–2 in the context of a *B. pseudomallei* infection. We abrogated *zip–2* in *ugt–29::GFP* reporter worms and assessed fluorescence intensity relative to control RNAi worms during a BpR15 infection. As shown in Fig. 4b, when ZIP–2 is compromised, the intense fluorescence in BpR15-infected worms was markedly reduced to an intensity comparable to basal expression of uninfected worms, proposing that ZIP–2 is required to mediate the induction of *ugt–29*. Quantification of *ugt–29* transcript levels by RT-PCR confirmed this reduction, further validating that *B. pseudomallei*-induced expression of *ugt–29* requires ZIP–2 (Fig. 4c).

### Induction of *ugt-29* is in response to inhibition of host protein synthesis

**Figure 5:**
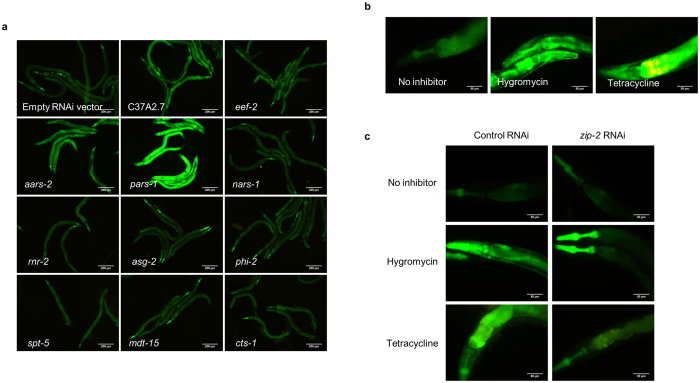
Translational inhibition induces *ugt-29* expression in the absence of infection. (**a**) Representative fluorescence micrographs (100× magnification) of *ugt–29::GFP* reporter worms grown on RNAi lawns that target and inactivate several individual components of essential processes in worms. Worms were exposed to the RNAi lawn for 48 hours starting at the L1 stage. Scale bar, 200 μm. (**b**) Representative fluorescence micrographs (400× magnification) of *ugt–29::GFP* reporter worms challenged with chemical protein synthesis inhibitors, 157 μg/ml hygromycin or 100 μg/ml tetracycline for 24 hours. Scale bar, 50 μm. (**c**) *ugt–29::GFP* worms were fed with RNAi clones of either the empty expression vector (control) or *zip–2* for 48 hours prior to treatment with 157 μg/ml of hygromycin or 100 μg/ml of tetracycline. The exposure time and gain factor are similar for all the micrographs taken at the same magnification. Scale bar, 50 μm.

The *zip–2* signalling pathway has recently been identified as a surveillance system utilized by worms to monitor the integrity of core cellular activities. Nematodes interpret any disruption of these activities as a consequence of pathogen attack and subsequently engage various defense responses which include aversion behaviour, detoxification and innate immune responses^21^,^22^,^23^. Interestingly, many of the critical cellular processes monitored by this cellular surveillance-activated detoxification and defense systems are known targets of bacterial toxins^22^. In light of its transcriptional regulation by ZIP–2, we asked if *ugt–29* is induced in response to cellular perturbations caused by *B. pseudomallei* toxins or virulence factors. To answer this, we performed RNAi-mediated gene inactivation on a panel of nematode genes known to encode core cellular components or metabolic enzymes (Table S1). L1 stage sterile *ugt-29::GFP* worms were fed with individual *E. coli* RNAi clones and the level of fluorescence was monitored 48 hours post-treatment. RNAi against four genes encoding translation factors (C37A2.7 or *eef–2*) and tRNA synthetases (*pars–1, aars–2*) resulted in significant over-expression of *ugt–29* relative to control RNAi-treated worms (Fig. 5a). Following RNAi knockdown, disruption of protein synthesis most likely triggered the induction of *ugt–29* even in the absence of infection. As translational inhibition is a common mode of toxic action, it is likely that *ugt–29* overexpression following a *B. pseudomallei* infection is a result of toxin-induced translational inhibition.

To validate this, we examined if microbial molecules known to inhibit host translation could cause overexpression of *ugt–29* in worms. *ugt–29::GFP* transgenic worms were exposed to protein synthesis inhibitors, hygromycin B^24^ (157 μg/ml) or tetracycline^25^ (100 μg/ml), for 24 hours and fluorescence was monitored. Worms treated with either inhibitor exhibited intense fluorescence in comparison to untreated control worms (Fig. 5b). This is in agreement with the observed inducible expression of *ugt–29* in worms whose protein translation machinery was disrupted by RNAi. We further examined if the induction of *ugt–29* by hygromycin and tetracycline is also ZIP–2-dependent. To address this, *zip–2*-deficient *ugt–29::GFP* worms were exposed to the inhibitors and fluorescence was assessed relative to control RNAi-treated worms. As expected, *zip–2* abrogation led to suppressed *ugt-29* expression upon supplementation with hygromycin B or tetracycline (Fig. 5c). *zip–2* RNAi worms exhibited only mild fluorescence that was comparable to basal level expression of *ugt–29* in untreated worms, although an intense GFP signal was noted in the pharynx of worms treated with hygromycin B. Altogether, these data suggest that the key event in a *B. pseudomallei* infection leading to *zip–2*dependent *ugt–29* induction is translation inhibition.

### Bactobolin is the toxic molecule that triggers overexpression of *ugt-29*

As *ugt–29* is most likely induced by the presence of toxic molecules, we determined if the *B. pseudomallei* BLF-1 toxin triggers the induction of *ugt–29* during a *B. pseudomallei* active infection. Following worm infection, we noted that the BLF1 mutant retained the capacity to strongly induce *ugt–29* (Supplementary Fig. S4). No noticeable reduction in fluorescence was observed between worms infected by the BLF–1 mutant and its isogenic wild type strain, K96243. This suggests that BLF1 does not contribute to or is not solely accountable for the strong induction of *ugt–29* observed in *C. elegans* infected with *B. pseudomallei*. This was a strong indication that other unidentified toxin(s) of *B. pseudomallei* are most likely involved in the activation of the *zip–2*/*ugt–29* surveillance pathway.

**Figure 6:**
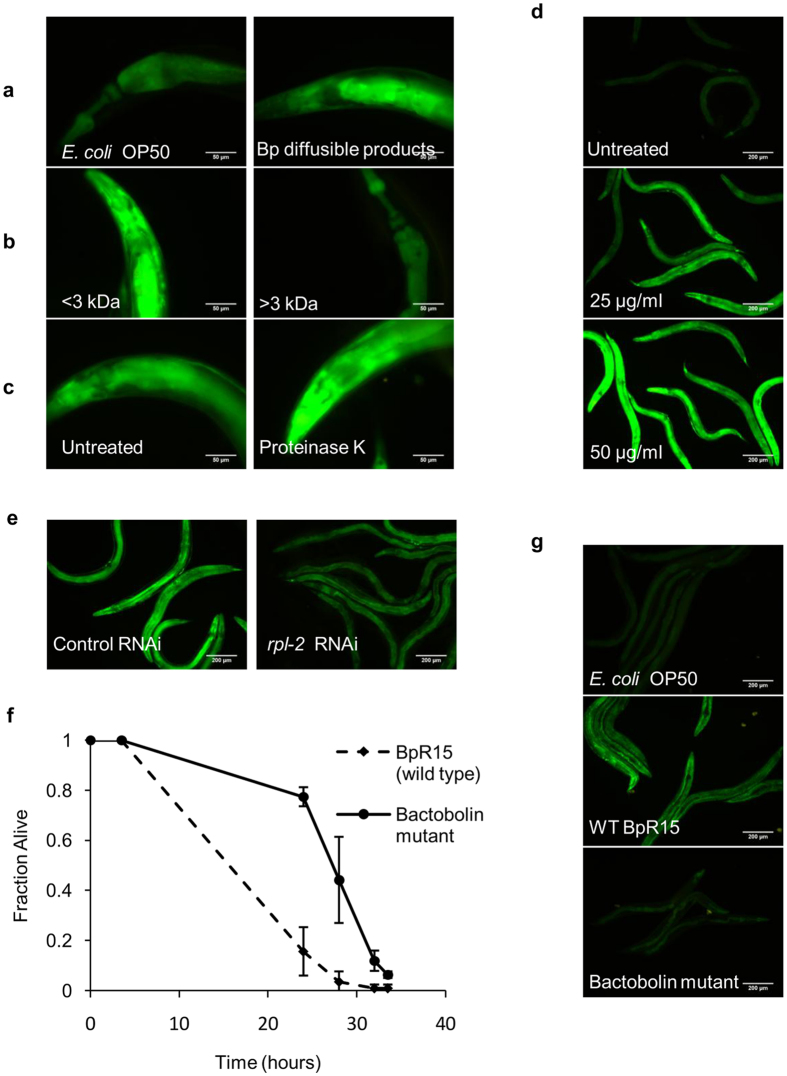
Bactobolin is the toxic molecule that triggers overexpression of *ugt-29*. Representative fluorescence micrographs (400× magnification) of *ugt–29::GFP* worms exposed to (**a**) *E. coli* OP50 or *B. pseudomallei* diffusible products, (**b**) <3 kDa or >3 kDa size fractions of BpR15 diffusible products and (**c**) untreated (control) or Proteinase K-treated BpR15 diffusible products for 24 hours. The *B. pseudomallei* compound that induces *ugt-29* is predicted to be an extracellular non-proteinaceous entity of <3 kDa. (**d**) Representative fluorescence micrographs of *ugt-29::GFP* worms (100× magnification) treated with 25 μg/ml or 50 μg/ml of bactobolin for 24 hours. (**e**) Representative fluorescence micrographs of *ugt–29::GFP* worms (100× magnification) fed with RNAi clones of either the empty expression vector (control) or *rpl–2* for 48 hours prior to treatment with 25 μg/ml of bactobolin. (**f**) Kinetics of *C. elegans* killing by the bactobolin mutant and its isogenic wild type BpR15. Shown is the representative of two independent experiments (n=120). (**g**) Representative fluorescence micrographs of *ugt–29::GFP* worms infected by the bactobolin mutant and its isogenic wild type BpR15 at 24 hours post-infection. Fluorescence micrographs of the same magnification were acquired at the same exposure time and gain factor. Scale bars, 200 μm (100×) and 50 μm (400×).

As bacterial toxins are usually secreted, the search for the potential toxic molecule(s) of *B. pseudomallei* that induces nematode *ugt–29* expression was undertaken on the premise that *ugt–29::GFP* worms would exhibit increased fluorescence when exposed to BpR15 secreted products. The bacterial secretome was able to promote fluorescence demonstrating that the substance(s) responsible for inducing *ugt-29* was indeed secreted extracellular (Fig. 6a). When the pooled diffusible products were size fractionated by membrane ultrafiltration, only worms challenged with the fractions of < 3 kDa exhibited strong fluorescence, implicating that the target substance(s) is of a size < 3 kDa (Fig. 6b). To ascertain if the target substance(s) is proteinaceous, the diffusible products were digested with proteinase K. We noted that the digested diffusible products were still able to induce strong *ugt–29* suggesting that the target substance(s) is not a protein (Fig. 6c) and is most likely a metabolite.

To enable the identification of *B. pseudomallei* metabolite(s) responsible for the induction of *ugt–29*, comparative metabolite profiling was conducted. As noted above, *B. cepacia* but not *B. thailandensis* shared a similar ability as *B. pseudomallei* to strongly induce *ugt–29*. Hence, this suggested that the target substance(s) is common to both *B. pseudomallei* and *B. cepacia* but not *B. thailandensis*. Non-targeted LC-MS (liquid chromatography-mass spectrometry) metabolite profiling was performed on the diffusible products of all three individual species and potential targets were shortlisted based on a comparison between all three profiles. Four or five biological replicates of the diffusible products of all three *Burkholderia* species were collected and profiled. The features (retention time and mass, m/z) derived from XCMS were first clustered using RamclustR to reduce dataset redundancy. For multivariate analysis, principal component analysis (PCA) indicated close clustering of metabolite data within replicates of each species and distinct separation among the species tested (Supplementary Fig. S5). As the LC-MS data was highly complex, an orthogonal partial least square-discriminant analysis (OPLS-DA) was employed to identify the compounds that most strongly contributed to the model of comparison and we obtained a shortlist of 36 compounds (Table 1). These compounds were annotated based on spectral matching to five different metabolite databases: an in-house small molecules library of the Proteomics and Metabolomics Core Facility (PMF) at Colorado State University, NISTv12, Golm, Metlin Mass Spectral database and Massbank metabolite databases. However, only five of the compounds had a match in the metabolite databases and were annotated with identities whilst the remaining compounds were resolved as unknowns or peptides. Among the annotated targets, the compounds with the highest fold change between *B. pseudomallei* or *B. cepacia* and *B. thailandensis* were identified as bactobolins i.e. bactobolin A, B and D.

Bactobolin is a family of polyketide-peptide hybrid molecules that act as broad-spectrum antibiotics and is cytotoxic to mouse fibroblast cells. Bactobolin targets the prokaryotic 50S ribosome-associated L2 protein and its homologue in eukaryotes, L8e, is presumed to be the conserved target^26^. To validate whether bactobolin is the bacterial compound that induces nematode *ugt–29* expression, we exposed *ugt–29::GFP* worms to 25 μg/ml or 50 μg/ml synthetic bactobolin A and fluorescence was monitored. As depicted in Fig. 6d, 25 μg/ml of bactobolin was sufficient to strongly induce the expression of *ugt–29* whilst the higher dose intensified the overexpression of this gene in worms. Hence, the toxic action of bactobolin most likely inhibits translation in *C. elegans* through the L8e protein resulting in overexpression of *ugt–29* in worms. To determine if bactobolin A targets L8e, we RNAi inactivated *rpl–2* (the gene encoding L8e) in *ugt–29::GFP* worms and monitored fluorescence intensity following bactobolin treatment relative to control RNAi worms. We propose that abrogation of the bactobolin cellular target would result in decreased *ugt–29* expression. As expected, control RNAi-treated *ugt-29::GFP* worms exhibited strong fluorescence upon supplementation with 25 μg/ml of bactobolin A (Fig. 6e). For *rpl–2* knockdown worms, a marked reduction in fluorescence intensity was noted in comparison to RNAi control worms. This suggests that the inducible expression of *ugt–29* during exposure to bactobolin is essentially dependent on functional RPL–2 protein and that bactobolin affects host protein biosynthesis, which in turn, leads to the overexpression of *ugt–29*.

A partial in-frame deletion of the bactobolin encoding gene in BpR15 (Δ*bpss1174*) was successfully generated, as verified by the amplified truncated sequence of *bpss1174* (Supplementary Fig. S6). The gene deletion rendered the mutant *B. pseudomallei* significantly less effective in killing the worms (Fig. 6f) with parallel suppression of *ugt–29* expression (Fig. 6g). The TD_mean_ of worms infected by the bactobolin mutant was significantly delayed when compared to that of worms infected by wild type BpR15 (*p* < 0.0001, log-rank test). In addition, a nematode lifespan assay demonstrated a dose-dependent reduction in the lifespan of worms supplemented with bactobolin A, further confirming the toxicity of this compound on worms (Supplementary Fig. S7). Taken together, these data verify that bactobolin is a toxic bacterial compound of *B. pseudomallei* and is responsible for the induction of *ugt–29*.

## Discussion

Lee *et al*.^4^ had previously demonstrated that *ugt–29* was the most robustly induced *C. elegans* phase II detoxification gene following *B. pseudomallei* infection of worms. In parallel with the reports that suggest *B. pseudomallei* pathogenicity is mediated by bacterial toxins^5^,^8^,^9^, we propose that the robust induction of selected worm detoxification genes is most likely a host response towards the presence of *B. pseudomallei* toxins. In this study, we constructed a reporter transgenic strain for *ugt–29* and demonstrated that *ugt–29* expression was specific to infections by *B. pseudomallei* and *B. cepacia*, both of which are similar in degree of virulence in the worm infection model. We also demonstrated that induction of *ugt–29* is ZIP–2-dependent and the *zip–2*/*ugt–29* activation suggests a role in surveillance of defective mRNA translation. Taken together, we speculate that the over-induced *zip–2*/*ugt–29* in *B. pseudomallei*-infected worms is indicative of the presence of a *B. pseudomallei* toxic molecule that interferes with host protein translation.

*C. elegans* possess a large arsenal of detoxification enzymes that are involved in microbial defense and xenobiotic biotransformation^27^. Several reports have disclosed evidence that *C. elegans* elicits different detoxification responses upon challenge by different xenobiotic onslaughts^28^,^29^,^30^,^31^. This implies a specific and distinct response for each detoxification protein-encoding gene which justifies the presence of a large number of these genes in the worm^27^. Our results support the specific response of detoxification enzymes where a strong induction of *ugt–29* was observed following infection by only two out of the eight pathogens tested. As *ugt–29* is highly induced by *B. pseudomallei* and not by the closely related but less virulent *B. thailandensis, ugt–29* is most likely required for defense against microbial pathogenic factors as supported by the correlation between strong induction of *ugt–29* with strong virulence capacity in killing nematodes.

The *zip–2* signaling pathway is an immune pathway that responds specifically to *P. aeruginosa*, in parallel with but independent of, several well-described signaling pathways e.g. the PMK–1 p38 MAPK and FSHR–1 pathways^20^. In this study, we demonstrated that *zip–2* is also activated during a *B. pseudomallei* infection and that ZIP–2 is essential for complete resistance against killing by *B. pseudomallei*. In fact, ZIP–2 target genes which include the cohort of detoxification genes (CYP–14A5, *pgp–*5, *pgp–7, ugt–31* and *ugt–*29) and infection response genes (*irg–1* and *irg–2*) were amongst the most robustly induced transcriptional suite in response to *B. pseudomallei* infection^4^. Interestingly, while *zip–2* is comparably active during nematode infection by *P. aeruginosa* or *B. pseudomallei, ugt–29* is strongly induced by *B. pseudomallei* but not by *P. aeruginosa*. This could be due to different pathogenic mechanisms adopted by these two pathogens. Generally, *P. aeruginosa* relies on bacterial colonization rather than intoxication during slow killing with toxins only playing a minor role, justifying the minimal activation of *ugt–29* during a *P. aeruginosa* infection^32^. On the other hand, *B. pseudomallei* does not utilize colonization as the pathogenic strategy in worms^8^.

The *zip–2* signaling pathway has been suggested to act as a surveillance mechanism for mRNA translation as well as other essential cellular processes as a means to discriminate pathogens from innocuous microbes. Worms are able to recognize translational inhibition by *P. aeruginosa* ToxA and go on to engage the *zip–2* defense^21^,^23^. Hence, the *zip–2*/*ugt–29* relationship could represent an effector-triggered immune (ETI) response during a *B. pseudomallei* infection. In this study, *zip–*2/*ugt–29* was shown to be responsive to cellular perturbations in mRNA translation, confirming the surveillance role of *zip–2*. As a highly virulent *B. pseudomallei* isolate could trigger remarkably strong *ugt–29* expression, an induction of *ugt–29* in *B. pseudomallei*-infected worms implicates a lethal toxin action. Together, these observations have paved the way to utilizing *ugt–29* expression as a readout of putative *B. pseudomallei* toxic molecules.

The finding that bactobolin strongly triggers *ugt–29* expression verifies the initial idea of utilizing *ugt–29* to sense for microbial compounds that are able to disrupt the host translation apparatus. Bactobolin inhibits the growth of bacterial and mammalian cells by inhibition of protein synthesis^26^,^33^. The molecular basis of translation inhibition by bactobolin was recently revealed as a consequence of a conformational rearrangement of P-site tRNA that interferes with translation termination^34^. While the toxicity of bactobolin from other bacteria has been extensively studied, our findings propose this small molecule as a new virulence attribute of *B. pseudomallei*. Bactobolin is only the second *B. pseudomallei* bacterial toxin identified to date and more interestingly, both bactobolin and BLF1^9^ exploit the host translation machinery to achieve the lethal effect, although targeting different components of the translational apparatus.

While several reports provide evidence for the production of bactobolin by *B. thailandensis*^26^,^35^,^36^, in this study, bactobolin is not secreted in significant amounts by *B. thailandensis*. One possible explanation is that the biosynthesis and secretion of bactobolin are not constitutive and remain cryptic throughout our experimental manipulation. Indeed, it was reported that *B. thailandensis* bactobolin is produced only at 30 °C but not at 37 °C^26^. The identification of bactobolin as the toxic small molecule also supports the prevalent role of secondary metabolites in bacterial pathogenesis. *P. aeruginosa* strains have been reported to kill worms through secondary metabolites e.g. cyanide and phenazines^37^,^38^,^39^ whilst *B. pseudomallei* siderophore, biosurfactant and proteasome inhibitor are essential to promote death in the context of a murine infection model^40^,^41^.

This is the first report on the utilization of a nematode biosensor to successfully identify a potent bacterial toxin. As *ugt–29* expression is triggered when the key translation machinery is disrupted, we suggest that the *ugt–29::GFP* construct is a useful tool for the identification of toxins, particularly those that target the host translational machinery. Our findings have also confirmed that *ugt–29* induction is not solely activated by a single xenocompound or bacterial toxin. In addition, hygromycin and tetracycline share the similar ability as bactobolin to trigger strong *ugt–29* induction and are also toxic to worms. Worms treated with hygromycin exhibit a significantly reduced lifespan whilst tetracycline negatively affects nematode growth and reproduction^23^,^42^.

In summary, we have shown that UGT–29 is part of the ZIP–2-mediated cellular surveillance pathway in response to defects within different factors associated with the host translation machinery. In the context of a *B. pseudomallei* infection, the robust increase in *ugt–29* expression suggests significant perturbations in protein synthesis, which is likely the consequence of potent bacterial toxins or virulence factors. Through the utilization of *ugt–29::GFP* worms and comparative LC/MS, we identified bactobolin as the toxic bacterial molecule that triggers *ugt–29* expression. As *ugt–29* expression implicates an overall subversion in translation, *ugt–29::GFP* may serve as a valid nematode biosensor for toxin identification.

## Methods

### Bacterial and *C. elegans* strains

The bacterial and worm strains used in this study are listed in Supplementary Table S2. All experiments involving *B. pseudomallei* were approved by Universiti Kebangsaan Malaysia Animal Ethics Committee (UKMAEC) and performed in a BSL2 + level laboratory. Growth and manipulation of *C. elegans* were performed as previously described^43^.

### Construction of the transgenic *ugt-29* *::GFP* expressing strain

A 964 – bp promoter fragment of the *ugt–29* gene (Wormbase ID: WBGene00021709) was amplified from *C. elegans* genomic DNA with primers UGTF1 (5′GAGAAGCATCTTTGGGCAATGGTCTA3′) and UGTR1 (5′AGTCGACCTGCAGGCATGCAAGCTAAAGAATTGAGTAGCATTGTGAAGT3′) in which the reverse primer was incorporated with 24–bp of the GFP coding sequence (underlined). Genomic DNA was prepared from individual animals using methods adapted from Barstead *et al*.^44^. The 1892–bp *Aequorea coerulescens gfp* ORF was amplified from pPD95.75 (a gift from Prof. Andrew Fire, Stanford University, USA) with the primers GFPF1 (5′*AGCTTGCATGCCTGCAGGTCG* 3′) and GFPR1 (5′AAGGGCCCGTACGGCCGACTA 3′). Using the PCR fusion technique previously described^45^, both amplicons were fused at the 24–bp overlapping region generated at the 5′ end of the *ugt–29* negative strand and *gfp* ORF positive strand (italicized sequence), then amplified with a set of nested primers, NUGTF1 (5′ATCTTTGGGCAATGGTCTAGACGAG 3′) and NGFPR1 (5′GGCCGACTAGTAGG AAACAGTTATG 3′), to form the *ugt–29::GFP* construct. High-fidelity *Pfu* DNA polymerase was used for all DNA amplifications. The resulting fusion product (50 ng/μl) was then microinjected along with 100 ng/μl pBX (*pha–1*^+^) (a gift from Prof. Andrew Fire, Stanford University, USA) as a coinjection marker into the gonadal syncytium of young adult stage *pha-1(e2123)ts* animals using a FemtoJet microinjector (Eppendorf AG). Transformed worms were selected by incubating them at 25  C prior to screening for GFP expression. The transformed worms were identified by observation of GFP expression using a Leica DMRXA2 upright fluorescence microscope equipped with a Leica I3 long-pass GFP filter (Leica Microsystems).

### GFP reporter experiments and microscopy imaging

Germline proliferation-deficient worms (Glp) were produced as previously described^17^ to avoid difficulty in GFP observation within transgenic worms (*ugt–29::GFP, hsp–16.2::GFP* and *pgp–5::GFP*) due to the spatial interference by nematode eggs. For every indicated experimental condition, ~ 100 Glp transgenic worms were transferred to the assay plates (pathogen-seeded, RNAi or chemical–supplemented plates) and kept at 25 °C until the completion of the assay. At every indicated time point, 10 to 15 transgenic worms were mounted on a 2% agarose pad prepared on a glass slide for GFP examination. Five microliter of 5 mM levamisole was used as the paralyzing agent. The worms were observed under 100× and 400× magnification using a Leica upright fluorescence microscope equipped with a Leica I3 long pass GFP filter (Leica Microsystems) and all micrographs were captured using the Leica DCF 310 FX digital camera and LAS version 3.8 software (Leica Microsystems).

### Pathogen infection experiments

To prepare the bacterial lawn, all species of *Burkholderia* including *B. pseudomallei* were cultured overnight at 37 °C in Brain Heart Infusion (BHI) broth (Pronadisa) and spread on Nematode Growth Media (NGM) plates. For *P. aeruginosa*, the culture was grown in King’s B broth supplemented with 100 μg/ml rifampicin at 37 °C and spread on SK (slow-killing) plates. For *S. aureus* experiments, an overnight culture grown in Trypticasein Soy broth (Pronadisa) at 37 °C was spread on Trypticasein Soy agar (Pronadisa) whilst for *E. faecalis*, bacteria grown overnight in BHIB at 37 °C were spread on BHI agar (Pronadisa). In all cases, 10 μl of the overnight culture was spread on a 3.5 cm assay plate while for the larger 6 cm assay plate, 30 μl of the overnight culture was spread. The assay plates were incubated at 37 °C for 24 hours, equilibrated at room temperature for 30 minutes to 24 hours before the worms were transferred for infection. Throughout the infection, assays plates were kept at 25 °C until completion of the assay. For heat-killed BpR15, 3 ml overnight culture was pelleted at 3 220 × *g* for 20 minutes. The pellet was resuspended in 100 μl BHIB and incubated at 95 °C for 10 to 15 minutes before seeding on a 6 cm NGM plate.

### RNAi interference

RNAi interference was performed by feeding the nematodes (Glp *ugt–29::GFP* transgenic worms for GFP examination or *rrf–3(pk1426);glp–4(bn2)* worms for survival assay) with *E. coli* strain HT115 (DE3) expressing double-stranded RNA homologous to the target gene of interest. The *E. coli* RNAi clone was grown in LB medium supplemented with 100 μg/ml carbenicillin at 37 °C overnight. The concentrated culture (25–fold) was seeded onto NGM agar containing 1 mM Isopropyl b-D-1-thiogalactopyranoside (IPTG) (Promega) and was left to dry at room temperature overnight. With the exception of *rpl–2*, L1 or L2 larval stage Glp worms were transferred to and raised on NGM plates seeded with the individual *E. coli* RNAi clone for 48 hours at 25 °C. *zip–2*-treated worms were subsequently exposed to *B. pseudomallei* infection or chemical treatments (157 μg/ml hygromycin or 100 μg/ml tetracycline). For *rpl–2*, a population of older-stage Glp worms (young adult woms) was exposed to the corresponding RNAi clone to avoid developmental arrest. After 48 hours of RNAi treatment at 25 °C, *rpl–2*-treated *ugt–29::GFP* worms were later exposed to treatment by 25 μg/ml or 50 μg/ml bactobolin. In all experiments, *E. coli* expressing an empty RNAi expression vector (L4440) served as the control. For chemical treatments, assay plates were prepared by adding the desired concentration of chemicals (157 μg/ml hygromycin, 100 μg/ml tetracycline, 25 μg/ml or 50 μg/ml bactobolin) to the molten NGM during agar preparation. Concentrated *E. coli* OP50 was added as the food source once the chemical-supplemented agars were solidified.

### *C. elegans* survival and lifespan assay

*rrf–3(pk1426);glp–4(bn2)* worms were synchronized and grown at 25 °C to adult stage. For infection assays, thirty age-matched worms were transferred to an assay plate seeded with pathogen (different isolates of *B. pseudomallei*, different *Burkholderia* species, *P. aeruginosa, E. faecalis* or *S. aureus*), three plates per strain for each experiment. For lifespan assessment upon treatment with bactobolin, thirty age-matched worms were transferred to NGM plates supplemented with 25 μg/ml or 50 μg/ml of synthetic bactobolin A and the experiment was performed in triplicate. *E. coli* OP50 was seeded on the NGM plates as food source. Survival of infected or treated worms was monitored over time until all the worms died. Worms were considered dead if they failed to respond to probing by a platinum wire picker. Worms that died of desiccation on walls were censored for further analysis. Statistical analysis of worm survival was performed using the Kaplan-Meier nonparametric analysis in the Statview software (version 5.0.1; SAS institute) whereby the statistical significance (*p*-values) was determined using the log-rank method.

### Environmental stress assays

Environmental stress assays were performed on transgenic worms as described^46^,^47^,^48^. Briefly, for the heat shock assay, sterile adult *ugt–29::GFP* transgenic worms were incubated at 37 °C for 1 hour and transferred to 25 °C over the assessment of GFP profile after heat stress^46^. To establish paraquat and cadmium toxicity assays, sterile adult *ugt–29*::*GFP* transgenic worms were transferred and maintained on NGM agar containing 10 mM paraquat^47^ or 100 μM cadmium^48^. All the experiments were carried out at 25 °C and for every stress condition, 10 to 15 worms were mounted and the GFP profile of stress-challenged worms was compared to untreated control worms at 6 (paraquat) and 24 hours post treatment (heat stress and cadmium). TJ375 (*hsp–16.2::GFP*) and WE5172 (*pgp–5::GFP*) transgenic worms were assayed in parallel as the control worms to ensure that the stress conditions were optimally established.

### Total RNA isolation and quantitative RT-PCR (qRT-PCR)

Age-matched sterile adult worms were transferred to pathogen-seeded assay plates for infection, after which, total RNA was isolated using Trizol reagent (Invitrogen) from the population of worms infected by different pathogens at 24 hours post infection and by different isolates of *B. pseudomallei* at 8 hours post infection. Total RNA extracted was further purified using Qiagen RNeasy columns (Qiagen) and DNase-treated using RNase-free DNase (Qiagen). qRT-PCR was performed on purified RNA using iScript^TM^ One-Step RT-PCR kit (BioRad Laboratories) with SYBR green detection on the CFX96 Touch Real-Time PCR Detection System (BioRad Laboratories). The *ugt–29* transcript level was measured using the forward primer (5′-TATATGCCAAAGAAATGGAGAAAC-3′) and the reverse primer (5′-CGAATACTGATAGTCGGGGATC-3′). Specificity of amplification was confirmed by melt curve analysis whereas amplification efficiency was determined by slope of standard curve. C_t_ (threshold cycle) values were accurately normalized by means of geometric averaging to three internal control genes (*ama-1,* F44B95 and pan actin)^4^,^49^. The alteration of *ugt-29* transcript level in infected worms was calculated relative to uninfected worms.

### Characterization of BpR15 diffusible products.

To obtain diffusible bacterial products, 30 μl of a 3 ml overnight culture of BpR15 was spread on 0.22 μm cellulose nitrate membrane filters (Whatman) on top of 6 cm NGM agar plates^17^. The plates were incubated at 37 °C for 24 hours after which the filter paper was removed. *E. coli* OP50 was seeded as the food source and *ugt–29::GFP* transgenic worms were transferred to the plates for GFP examination at 24 hours post exposure. To further determine the size range of the bacterial product(s) that induces *ugt–*29, three plates of NGM agar with embedded diffusible bacterial products were soaked with 4 ml of sterile distilled water each for 24 hours and pooled together. The pooled diffusible products were size fractionated using Amicon Ultra-15 Centrifugal Filter units (Merck) with a molecular weight cut-off of 3 kDa. The fractionated samples were concentrated in the Eppendorf Concentrator plus^TM^ (Eppendorf) before spreading and drying on NGM agar. Concentrated *E. coli* OP50 was seeded as the food source and *ugt–29::GFP* transgenic worms were transferred to the plates for 24 hours before the GFP examination. For proteinase K digestion, 166 μg/ml proteinase K (Qiagen) was added to the pooled and concentrated sample fraction of <3 kDa. The sample was incubated at 55 °C for 1.5 hours in parallel with an untreated sample (without proteinase K) that served as a control. The proteinase K-treated and untreated samples were deposited on agar, followed by the seeding of *E. coli* OP50 as food source. *ugt–29::GFP* transgenic worms were subsequently exposed for 24 hours before GFP examination.

### Metabolite extraction for LC-MS

NGM plates with embedded diffusible products were prepared as described above. For each biological replicate bacterial sample (*B. pseudomallei, B. cepacia* and *B. thailandensis*), three plates of NGM agar were soaked with 4 ml of methanol-water mixture (MW; 1:1, v/v) for 24 hours and the mixture was pooled together. The pooled samples were concentrated in an Eppendorf Concentrator plus^TM^ (Eppendorf) for a complete dry down in order to avoid metabolite degradation, after which, the samples were sent to The Proteomics and Metabolomics Facility (PMF) at Colorado State University for LC-MS analysis. In brief, compounds were annotated based on spectral matching to the PMF, NISTv12, Golm, Metlin Mass Spectral database and Massbank metabolite databases. The peak areas for each feature in a spectrum were condensed via the weighted mean of all features in a spectrum into a single value for each compound. Analysis of variance (ANOVA) was conducted on each compound using the aov function in R, and *p*-values were adjusted for false positives using the p.adjust in R^50^. PCA was conducted on mean-centered and Pareto-scaled data using the pcaMethods package in R.

### Construction of the bactobolin mutant (Δbpss1174)

A partial in-frame deletion mutant of bactobolin in BpR15 was constructed according to the method described by Lopez *et al*.^51^ Briefly, two short fragments corresponding to the upstream and downstream sequences of *bpss1174* were amplified from BpR15 genomic DNA (Supplementary Fig. S7a). The primer pair used for amplifying the upstream sequences was US-F (5′-GACCCCGCCTATAACAATCC-3′) and US-R (5′-GAGTCCTCACCAGCACGAAC-3′) whilst the primer pair for the downstream amplification was DS-F (5′GTTCGTGCTGGTGAGGACTCCCCGGTTTTTCAGGCG TTTT-3′) and DS-R (5′-AAGATTGCGTGGTCAGGG-3′). The two amplified fragments were subsequently fused and cloned into the non-replicative plasmid pEXKm5 before transforming into *E. coli* mobilizer strain RHO3. The recombinant plasmid harboring the *bpss1174* gene fragment was introduced into BpR15 by conjugation and homologous recombination resulting in the formation of either a wild type or mutant strain with the latter counter selected using yeast extract tryptone (YT) agar containing 15% sucrose. The double-crossover clones were further screened by kanamycin selection and colony PCR. Further PCR and DNA sequencing were performed using the V-F (5′- AGTTACTGATGCGAAGGGGCCAA-3′) and V-R (5′-AGGTTGTTCAGCAGATAGTGCGGG-3′) primer pair flanking outward from *bpss1174* to confirm the successful generation of the bactobolin mutant.

## Additional Information

**How to cite this article**: Wong, R.-R. *et al*. Detection of *Burkholderia pseudomallei* toxin-mediated inhibition of protein synthesis using a *Caenorhabditis elegans ugt-29* biosensor. *Sci. Rep.* **6**, 27475; doi: 10.1038/srep27475 (2016).

## Supplementary Material

Supplementary Information

## Figures and Tables

**Table 1:** Identification and annotation of *B. pseudomallei* (BP), *B. cepacia* (BC) and *B. thailandensis* (BT) bacterial compounds by LC-MS.

**ID**	**Compound annotation**^a^	**Retention time (min)**	**Abundance of compound**^b^			**Fold change of abundance**^c^	
			**BT**	**BC**	**BP**	**BC vs BT**	**BP vs BT**
C24	bactobolin A	3.45	9	1046	647	115.62	71.58
C19	bactobolin B	3.61	11	654	185	60.41	17.06
C73	bactobolin D	3.95	4	313	209	70.36	46.99
C414	glabrin D, putative	3.37	185	507	547	2.74	2.96
C258	istamycin KL1	2.66	265	828	298	3.13	1.13
C107	peptide	0.67	38	542	531	14.15	13.86
C26	peptide	1.38	275	691	255	2.51	0.93
C342	peptide	3.21	156	390	417	2.50	2.68
C82	peptide	4.19	31	145	119	4.73	3.88
C378	peptide	1.68	152	774	260	5.11	1.72
C738	peptide	1.77	534	1151	348	2.16	0.66
C1486	peptide	1.79	1074	1553	1529	1.45	1.42
C102	peptide	2.59	243	482	409	1.99	1.68
C835	peptide	2.97	121	314	167	2.61	1.38
C1628	peptide	3.06	146	432	251	2.96	1.72
C1036	peptide	1.94	476	772	931	1.63	1.95
C1496	peptide	1.48	190	344	326	1.81	1.71
C1237	peptide	1.76	305	576	598	1.90	1.96
C59	similar to deoxypyridinoline	3.58	165	451	564	2.74	3.42
C90	unknown	2.89	286	1214	1331	4.25	4.66
C3	unknown	6.02	25	869	782	35.16	31.65
C94	unknown	0.76	92	508	289	5.54	3.15
C65	unknown	1.07	139	484	170	3.49	1.22
C36	unknown	2.83	1652	4167	1745	2.52	1.06
C173	unknown	2.83	263	685	630	2.61	2.40
C343	unknown	3.11	99	574	235	5.81	2.38
C495	unknown	3.58	54	349	100	6.42	1.85
C99	unknown	3.77	448	905	801	2.02	1.79
C195	unknown	2.75	171	353	270	2.06	1.58
C504	unknown	2.79	168	737	396	4.38	2.35
C1340	unknown	3.16	170	535	267	3.14	1.57
C1209	unknown	2.13	168	290	446	1.73	2.65
C260	unknown	2.52	109	315	479	2.87	4.38
C1423	unknown	2.96	649	775	904	1.19	1.40
C325	unknown	3.95	102	203	240	1.99	2.35
C524	unknown	1.74	307	801	754	2.61	2.46

^a^The compounds were identified based on mass spectral matching to available databases.

^b^Relative quantity of each compound was determined by calculating the mean area of the chromatographic peak.

^c^Fold change in compound abundance of *B. pseudomallei* or *B. cepacia* was calculated relative to *B. thailandensis*.
