# Supplementary material for: Detection of Burkholderia pseudomallei toxin-mediated inhibition of protein synthesis using a Caenorhabditis elegans ugt–29 biosensor
Source: Sci Rep. 2016 Jun 7;6:27475. doi: 10.1038/srep27475 (PMC4895344; doi:10.1038/srep27475)
Supplement: Supplementary Information [file srep27475-s1.pdf]

Supplementary Information

Detection of *Burkholderia pseudomallei* Toxin-Mediated Inhibition of Protein Synthesis Using a *Caenorhabditis elegans ugt-29* Biosensor

Rui-Rui Wong, Cin Kong, Song-Hua Lee & Sheila Nathan

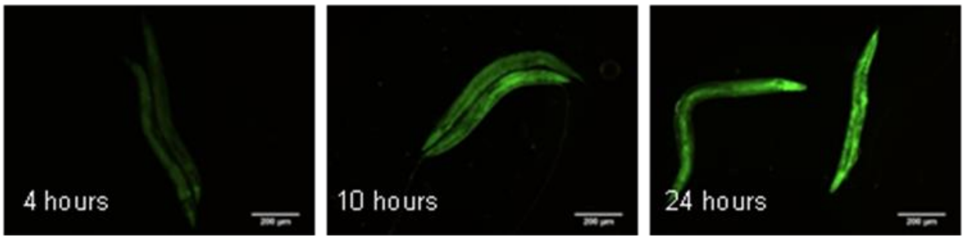

Figure S1. Representative fluorescence micrographs of BpR15-infected worms captured at 4, 10 and 24 hours post infection. Expression of *ugt-29* was progressively induced throughout the period of infection by BpR15.

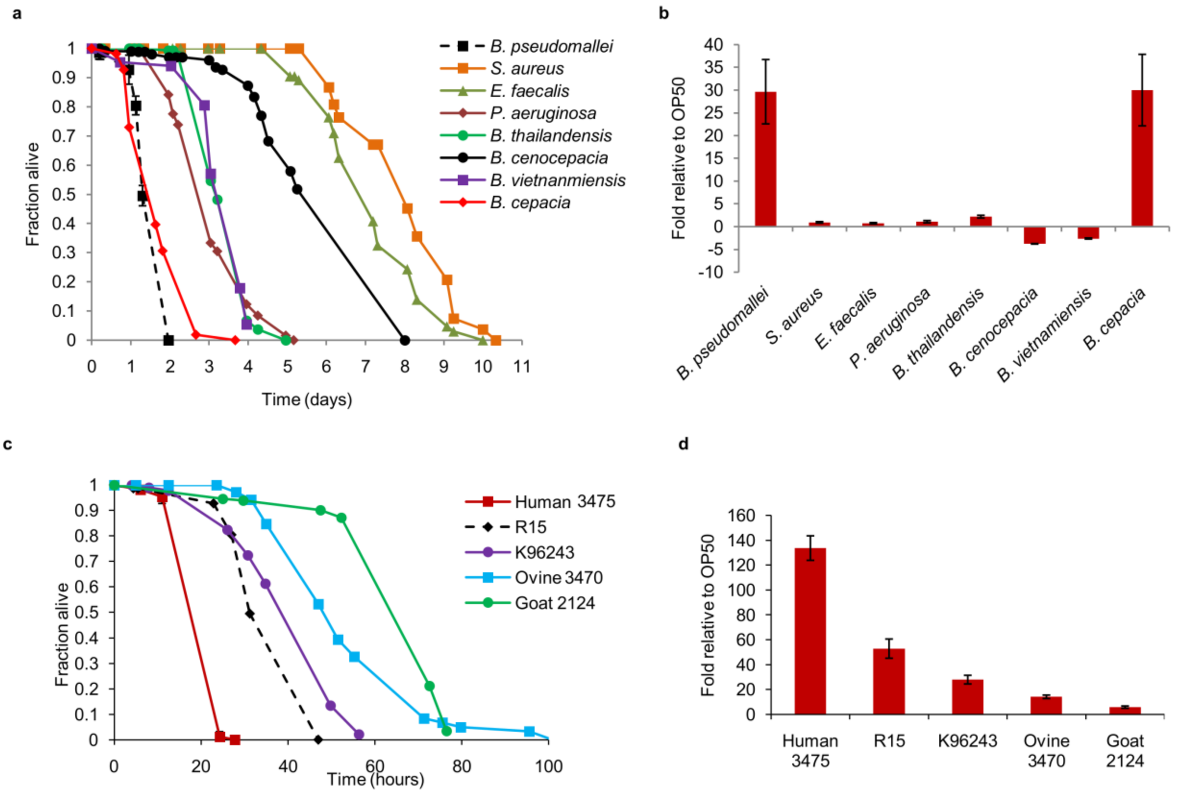

Figure S2. *ugt-29* expression is pathogenicity-related. (a) Kinetics of *C. elegans* killing by various pathogens. Shown is the representative of two independent experiments (n=120). (b) qRT-PCR analysis of *ugt-29* in response to various pathogens at 24 hours post infection. Error bars represent mean values ± SD. (c) Kinetics of *C. elegans* killing by different *B. pseudomallei* isolates. Shown is the representative of two independent experiments (n=120). (d) qRT-PCR analysis of *ugt-29* in response to different *B. pseudomallei* isolates at 8 hours post infection. Error bars represent mean values ± SD.

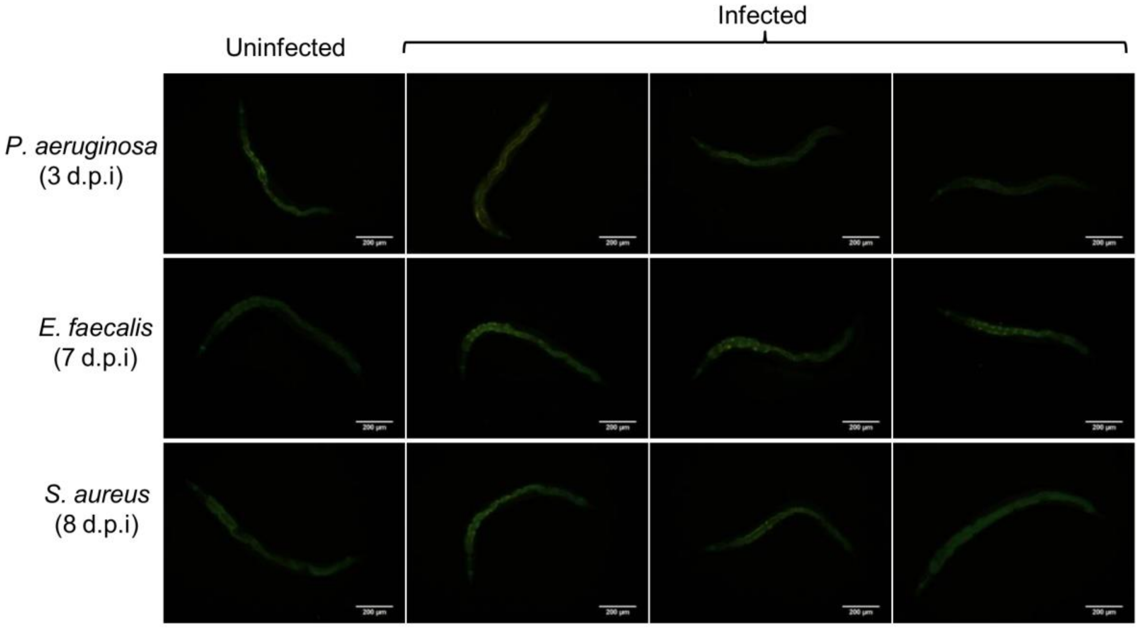

Figure S3. *ugt-29* is not induced even at a late stage of infection by *P. aeruginosa*, *E. faecalis* and *S. aureus*. GFP examination was extended to a time point at which 50% of the infected worms were killed. For each pathogen, three micrographs showing three representative infected worms are shown. Scale bars represent 200 µm. d.p.i is an abbreviation for days post infection.

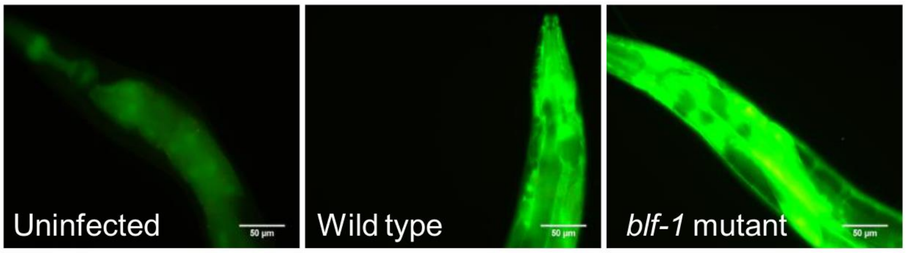

Figure S4. Suppression of BLF1 toxin production by *B. pseudomallei* does not reduce *ugt-29* overexpression in worms. Representative fluorescence micrographs (400× magnification) of *ugt-29::GFP* exposed to *E. coli* OP50 (uninfected), wild type K96243 and K96243 deficient in BLF1 production (*blf-1* mutant) for 24 hours. Micrographs were taken using a similar exposure time and gain factor. Scale bar, 50 µm.

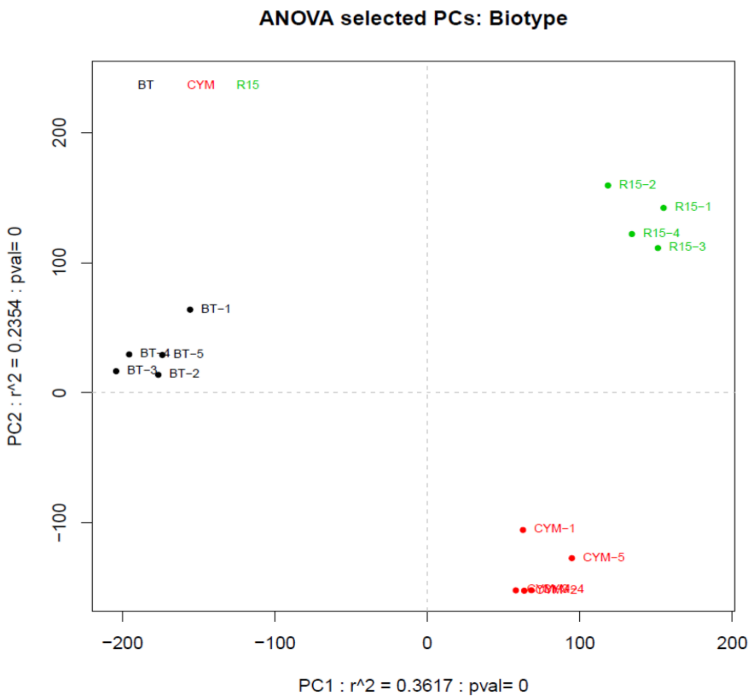

Figure S5. PCA plot of untargeted LC-MS metabolite analysis of *B. pseudomallei* (green), *B. cepacia* (red) and *B. thailandensis* (black). The plot demonstrates significant clustering of groups based on species and closed clustering between replicates within each species.

a

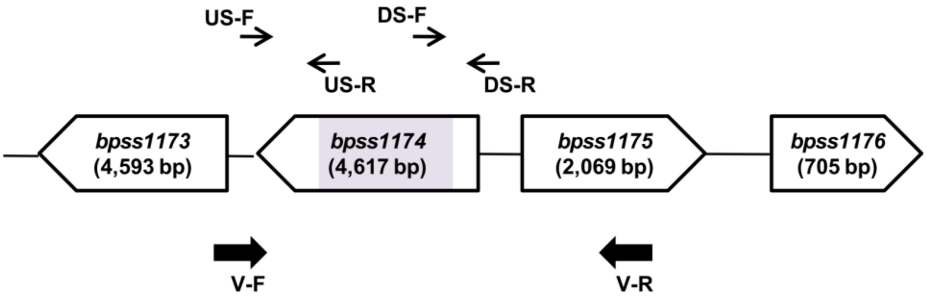

b

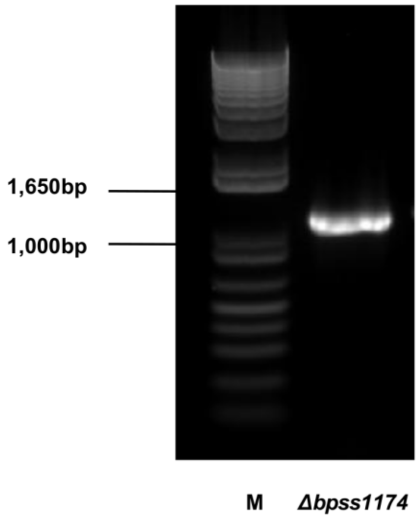

Figure S6. Construction of the bactobolin mutant (Δ*bpss1174*). (a) Gene organization of the partial gene cluster of *B. pseudomallei* for bactobolin biosynthesis. Primer binding sites are indicated by arrows. Highlights indicate the region deleted in the bactobolin mutant. (b) PCR analysis of Δ*bpss1174* using the primer pair, V-F and V-R. A DNA fragment of 1,063 bp was amplified, indicating the successful knockout of *bpss1174* gene in BpR15. M: Invitrogen 1Kb Plus DNA ladder.

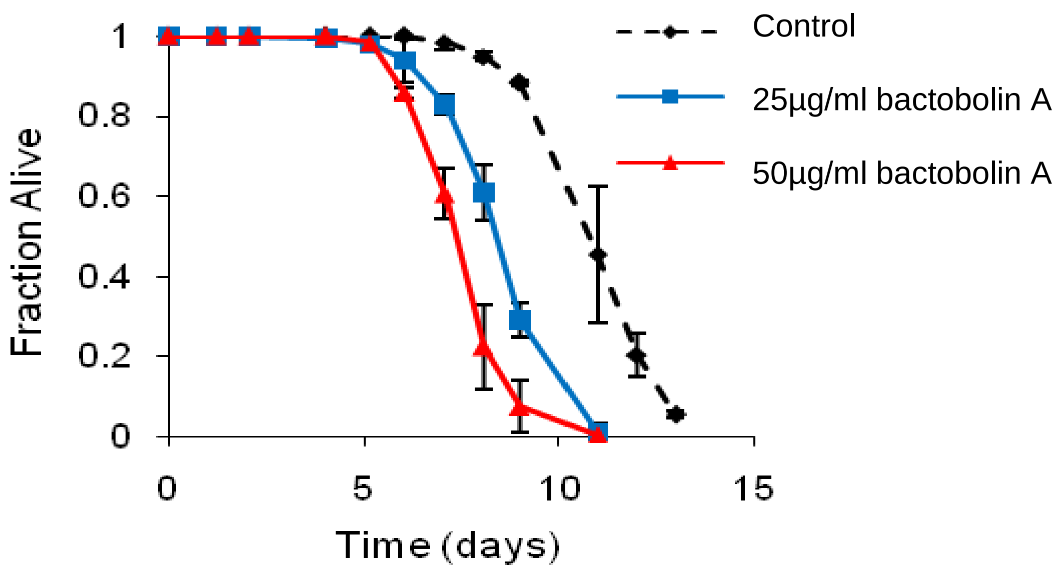

Figure S7. Lifespan of worms treated with 25 µg/ml or 50 µg/ml bactobolin. Both treatment doses significantly shortened lifespan relative to untreated control worms ($p < 0.0001$, log-rank test). Error bars represent mean value ± SD. Shown is the representative of two independent experiments (n=120).

Table S1. Genes selected for RNAi gene inactivation.

**RNAi target**	**Description**
**<u>Translation factor</u>**	
C37A2.7	Ribosomal protein 60S domain
eef-2	Translation elongation factor 2 (EF-2)
**<u>tRNA synthetase</u>**	
aars-2	Predicted cytoplasmic alanyl-tRNA synthetase
nars-1	Asparaginyl-tRNA synthetase
pars-1	Predicted cytoplasmic prolyl-tRNA synthetase
**<u>Ribonucleotide reductase</u>**	
rnr-2	Ribonucleotide reductase
**<u>ATP synthase</u>**	
asg-2	ATP Synthase G homolog
**<u>RNA polymerase II transcription elongation factor</u>**	
spt-5	RNA polymerase II transcription elongation factor
**<u>Fatty acid homeostasis regulator</u>**	
mdt-15	Mediator subunit orthologous to human MED15
**<u>Metabolic enzyme</u>**	
cts-1	Citrate synthase, predicted to be mitochondrial, TCA cycle

Table S2. Bacterial and worm strains used in this study.

Bacterial/nematode strains	Description	References
Burkholderia pseudomallei		
BpR15	Wild type clinical strain	(1)
K96243	Wild type clinical strain	(2)
Human 3475	Wild type clinical strain	This study
Ovine 3470	Wild type animal strain	This study
Goat 2124	Wild type animal strain	(1)
blf-1 mutant	K96243 strain defective in *blf-1* (a gift from Prof. Dr. Richard Titball)	(3)
Bactobolin mutant	BpR15 strain defective in *bpss1174*	This study
***Burkholderia* species**		
Burkholderia cepacia strain CYH	Species of *Burkholderia* cepacia complex (BCC)	(1)
Burkholderia vietnamiensis strain K2	Species of *Burkholderia* cepacia complex (BCC)	(1)
Burkholderia cenocepacia strain J2315	Species of *Burkholderia* cepacia complex (BCC)	(4)
Burkholderia thailandensis strain ATCC 700388	*Burkholderia* species which closely resembles *B. pseudomallei*	(5)
**Other pathogens**		
Pseudomonas aeruginosa strain PA14	Gram-negative pathogen	(6)
Staphylococcus aureus	Gram-positive pathogen	(7)

strain NCTC8325-4		
Enterococcus faecalis strain V583	Gram-positive pathogen	(8)
<u>Escherichia coli</u>		
Escherichia coli strain OP50	Normal laboratory food for *C. elegans*	(9)
E. coli strain HT115 expressing dsRNA directed against the *cdc-25.1*, *zip-2*, *eef-2*, *aars-2*, *pars-1*, *nrs-1*, *rnr-2*, *asg-2*, *phi-2*, *spt-5*, *mdt-15*, *cts-1*, C37A2.7 or *rpl-2*	RNAi clones for a targeted gene knock-down	Obtained from Geneservice and Open Biosystem, (10–12)
<u>Caenorhabditis elegans</u>		
Bristol N2	Wild type nematode	*Caenorhabditis* Genetics Center (CGC), (9)
rrf-3(pk1426);glp-4(bn2)	Contains loss-of-function mutations in the *rrf-3* and *glp-4* genes; exhibits germ line proliferation defect at 25°C and enhanced RNAi sensitivity	(13, 14)
TJ375	Transcriptional fusion; contains an integrated transgene *hsp-16.2::GFP*	(15)
WE5172	Transcriptional fusion; contains an integrated transgene *pgp-5::GFP*	(16)
ugt-29::GFP	Transcriptional fusion; *pha-1* mutant worm contains an extrachromosomal transgene *ugt-29::GFP*	This study

References

1. Lee, S. H. *et al. Burkholderia pseudomallei* suppresses *Caenorhabditis elegans* immunity by specific degradation of a GATA transcription factor. *Proc. Natl. Acad. Sci. U. S. A.* **110,** 15067–15072 (2013).
2. Holden, M. T. G. *et al.* Genomic plasticity of the causative agent of melioidosis, *Burkholderia pseudomallei*. *Proc. Natl. Acad. Sci. U. S. A.* **101,** 14240–14245 (2004).
3. Cruz-Migoni, A. *et al.* A *Burkholderia pseudomallei* toxin inhibits helicase activity of translation factor eIF4A. *Science* **334,** 821–824 (2011).
4. Holden, M. T. G. *et al.* The genome of *Burkholderia cenocepacia* J2315, an epidemic pathogen of cystic fibrosis patients. *J. Bacteriol.* **91,** 261–277 (2009).
5. Brett, P. J., Deshazer, D. & Woods, D. E. Characterization of *Burkholderia pseudomallei* and *Burkholderia pseudomallei*-like strains. *Epidemiol. Infect.* **118,** 137–148 (1997).
6. Tan, M. W., Mahajan-Miklos, S. & Ausubel, F. M. Killing of *Caenorhabditis elegans* by *Pseudomonas aeruginosa* used to model mammalian bacterial pathogenesis. *Proc. Natl. Acad. Sci. U. S. A.* **96,** 715–720 (1999).
7. Sifri, C. D., Begun, J., Ausubel, F. M. & Calderwood, S. B. *Caenorhabditis elegans* as a model host for *Staphylococcus aureus* pathogenesis. *Infect. Immun.* **71,** 2208–2217 (2003).
8. Garsin, D. A. *et al.* A simple model host for identifying Gram-positive virulence factors. *Proc. Natl. Acad. Sci. U. S. A.* **98,** 10892–10897 (2001).
9. Brenner, S. The genetics of *Caenorhabditis elegans*. *Genetics* **77,** 71–94 (1974).
10. Kamath, R. S. & Ahringer, J. Genome-wide RNAi screening in *Caenorhabditis elegans*. *Methods* **30,** 313–321 (2003).
11. Fraser, A. G. *et al.* Functional genomic analysis of *C. elegans* chromosome I by systematic RNA interference. *Nature* **408,** 325–330 (2000).
12. Rual, J. F. *et al.* Toward improving *Caenorhabditis elegans* phenome mapping with an ORFeome-based RNAi library. *Genome Res.* **14,** 2162–2168 (2004).
13. Shapira, M. *et al.* A conserved role for a GATA transcription factor in regulating epithelial innate immune responses. *Proc. Natl. Acad. Sci. U. S. A.* **103,** 14086–14091 (2006).
14. Simmer, F. *et al.* Loss of the putative RNA-directed RNA polymerase RRF-3 makes *C. elegans* hypersensitive to RNAi. *Curr. Biol.* **12,** 1317–1319 (2002).

15. Rea, S. L., Wu, D., Cypser, J. R., Vaupel, J. W. & Johnson, T. E. A stress-sensitive reporter predicts longevity in isogenic populations of *Caenorhabditis elegans*. *Nat. Genet.* **37,** 894–898 (2005).
16. Kurz, C. L., Shapira, M., Chen, K., Baillie, D. L. & Tan, M. W. *Caenorhabditis elegans pgp-5* is involved in resistance to bacterial infection and heavy metal and its regulation requires TIR-1 and a p38 map kinase cascade. *Biochem. Biophys. Res. Commun.* **363,** 438–443 (2007).
